# Unexpected Damage on Metal Artifacts Triggered by the Hazardous Interfacial Interaction from Aging of Polymer Coatings

**DOI:** 10.1021/acscentsci.5c00067

**Published:** 2025-04-23

**Authors:** Ying An, Pei Hu, Kaitao Li, Yu Kang, Gang Hu, Rui Tian, Chao Lu, Xue Duan

**Affiliations:** † State Key Laboratory of Chemical Resource Engineering, 47832 Beijing University of Chemical Technology, Beijing 100029, China; ‡ Pingyuan Laboratory, College of Chemistry, Zhengzhou University, Zhengzhou 450001, China; § Quzhou Institute for Innovation in Resource Chemical Engineering, Quzhou 324000, China; ∥ School of Archaeology and Museology, 12465 Peking University, Beijing 100871, China

## Abstract

Polymer coatings are currently being employed for preservation of metal artifacts. However, there is insufficient awareness that inevitable aging of polymer coatings is bound to damage metal artifacts due to the lack of a direct and sensitive methodology to study the aging behaviors of polymers coated on the artifacts. Herein, we have developed an in situ and three-dimensional strategy to visualize the early stage aging behaviors of polymers coated on metal artifacts by lighting carboxyl groups generated from polymer aging. It is disclosed that polymer aging occurred simultaneously at the surface and the interface with metal artifacts, generating carboxyl groups and hydroxyl radicals to induce the corrosion and oxidation of metallic artifacts. In turn, the generated metallic ions could further aggravate the aging of polymer coatings, manifested as the larger volume of the aged sites at the interface with metal artifacts in comparison with that at the surface of polymer coatings. Such a circular reaction is validated using real metal artifact samples. These findings raised a timely alarm for the conservation ability and potential threat of polymer coatings on metal artifacts. It is anticipated that the proposed strategy could provide solid supports for the implementation of advanced conservation strategies for metal artifacts.

## Graphical abstract

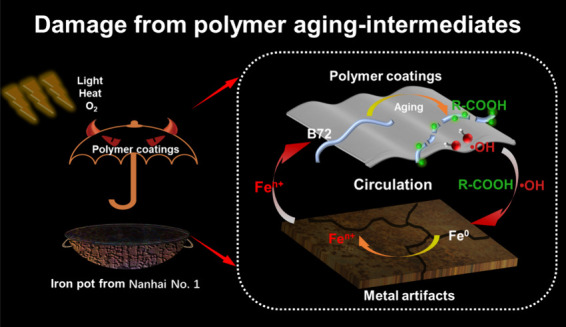

## Introduction

Metal artifacts are regarded as significant heritages due to their ancient roles in weapon manufacturing, labor tools, and commodity circulation.^1^ As the major ingredient of metal artifacts, iron element is apt to undergo serious erosion in a quite fast manner due to its active chemical properties.^2–4^ Oxidation and electrochemical corrosion primarily occur for the metal artifacts, especially under the atmosphere with oxygen and acidic medium.^5–7^ Therefore, it is highly necessary to conserve metal artifacts for avoiding the irreparable damages and immeasurable losses.^8,9^ Currently, polymers have been extensively employed as excellent candidates to preserve metal artifacts as coatings, water repellents, and consolidants owing to their light weight, strong adhesion, high strength, and excellent hydrophobicity.^10,11^ Generally, epoxy, polyurethane, and acrylic polymers are favorable in the conservation works for metal artifacts.^12–14^ It is believed that the deterioration of polymer-coated metal artifacts from external stimuli (e.g., heat, water, oxygen, and light) could be greatly reduced so that they would be reliably passed on to the future generations.^15,16^ However, the aging of polymers would naturally occur under these external stimuli, and thus it is inevitable to invalidate the reliability and protective ability of polymers.^17–19^

Polymer aging could generate different reactive oxygen radicals (e.g., alkoxy, peroxy, and hydroxyl radicals), and a series of aging products (acids, alcohols, ketones, and esters).^20–22^ Noticeably, metals are apt to be oxidized or corroded in the acidic environment, and such a process could be accelerated in the atmosphere with the aid of reactive oxygen radicals.^23,24^ Accordingly, we have reasons to believe that the irresistible polymer aging reactions would inevitably destruct metal artifacts through the interfacial interactions. To study the aging behaviors of polymers, traditional techniques were generally based on nuclear magnetic resonance, Fourier transform infrared spectroscopy, or gel permeation chromatography measurements, and the coated polymers should be first peeled off from the substrate or dissolved in a solvent.^25^ These procedures failed to provide a direct and in situ evaluation of the polymers coated on an artifact, lacking study of the interfacial interaction between polymer and artifact. Therefore, it is of great emergency to unravel how the aging of polymer coatings affect metal artifacts during the conservation process.

Three-dimensional fluorescence imaging has been proposed as a visual and spatial strategy through sequential sliced images and a three-dimensional reconstruction technique.^26^ It could provide ultrasensitive monitoring and quantitative analysis of the variations of the surface and internal structure of materials.^27,28^ On account of these advantages, we reasonably predict the application possibility of the fluorescence imaging technology in such an urgent problem of metal artifacts preservation. In this contribution, taking metal artifacts coated with acrylic resin B72 (the most widely used polymer in metal artifacts) as a typical example, the early stage aging behaviors of B72 on metal artifacts were investigated in situ by monitoring the variations of the carboxyl groups generated from the aging of B72 in a three-dimensional approach. It is noteworthy that the aging of B72 to generate carboxyl groups occurred not only at the surface but also at the interface with metal artifacts. Simultaneously, reactive hydroxyl radicals (^•^OH) were generated during the chain-broken process of B72. These active intermediates provided an acidic and oxidizing microenvironment for metal artifacts, leading to the corrosion of metal artifacts as a result of the oxidation of iron to generate iron ions (Scheme 1). Moreover, the quantitative results for the aged polymer coatings pointed out that the volume of the aged sites at the interface with metal artifacts was larger than that at the surface. Such an aggravated interfacial aging reaction could be ascribed to the facilitated chain scission of B72 by the metallic ions in the metal artifacts. Accordingly, a hazardous circulation was formed between B72 and metal artifacts, demonstrating the destructive threats from the aging of B72 during the conservation of metal artifacts. These findings were further validated on the iron debris from the Nanhai No. 1 and iron coin from the Northern Song Dynasty. Our findings have raised the alarms for the conservation of the metal artifacts. It is anticipated that the proposed strategy could be further extended to unravel the conservation state and potential risks for other artifact preservation, minimizing the damage to the valuable artifacts.

**Scheme 1:**
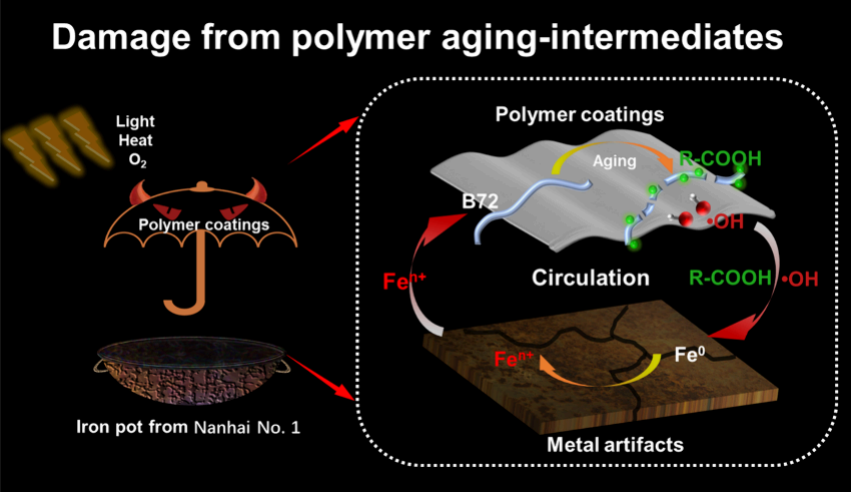
Schematic Representation for Polymer Coatings Damaging Metal Artifacts^a^

a Schematic representation of damage from the aged polymer on the metal artifacts. The enlarged image shows the interaction between polymer coatings and metal artifacts.

## Results and Discussion

### Visualized Aging Intrusion of B72

Paraloid B72 was a binary copolymer of methyl acrylate and ethyl methacrylate, and carboxyl groups could be formed in B72 through the chain breaking and oxidation after the photothermal aging.^29^ To visualize the aging sites of B72, we have employed 6-amino-fluorescein (AF) with an amino group as the fluorescence probe to label the generated carboxyl groups in the presence of *N*-hydroxysuccinimide (NHS) and 1-(3-(dimethylamino)­propyl)-3- ethylcarbodiimide hydrochloride (EDC).^30^ First, we have explored the ability of AF molecules to label carboxyl groups employing sodium polymethacrylate (PMAA) with abundant carboxyl groups. The contents of carboxyl groups in PMAA were determined by potentiometric titration (Figures S1A–S1E and Table S1). The fluorescence changes of AF (Δ*I* = *I* – *I*_0_) showed a good linear relationship with the increased concentration of carboxyl group (*c*): Δ*I* = 12.82*c* + 133.02 (*R* ^2^ = 0.9939, Figure S1F), suggesting that the AF could accurately label the carboxyl groups in polymers. B72 was coated on cast iron, and the thickness of B72 was measured by a thickness gauge. Subtracting the thickness of the bare cast iron (0.734 mm) from the total thickness of the B72/Fe (0.749 mm), the thickness of B72 was calculated to be approximately 15 μm (Figure S2). Second, the photothermally aged B72/Fe was labeled by AF and studied through the three-dimensional fluorescent imaging strategy. Bright green fluorescence sites could be observed for the aged B72/Fe after labeling in the AF, while the unlabeled B72/Fe showed clear background under the same excitation light of 488 nm (Figure S3A). Therefore, we could utilize the AF to target the generated carboxyl groups and visualize their variations through the fluorescence imaging strategy.

To simulate the conservation condition of immovable cultural artifacts that were exposed under sunlight and high temperature,^31^ B72/Fe was treated under photothermal conditions with the temperature of 60 °C and UV irradiation of 1.0 W/m^2^. Three-dimensional fluorescence images were captured for B72/Fe after photothermal treatment for different time (Figure 1A). It was found that the unaged B72/Fe showed no fluorescence signals (Figure S3B). In contrast, obvious fluorescence sites appeared in the B72/Fe after photothermal treatment. With the prolonged treatment time from 3 to 30 h, the fluorescence sites grew continuously larger (Figures S3C–S3J), suggesting the aggravated aging status for B72 on the cast iron. Notably, the lighted aging sites showed pyramidal or needlelike morphologies in the B72 on the cast iron (Figure 1B), which could be ascribed to the perforative aging along the vertical direction through the B72 of ∼15 μm. Such a phenomenon could be identified by the side-view images of the aged B72/Fe (Figure 1C). Surprisingly, fluorescence sites appeared from the both sides of the B72: one side from the surface in contact with the air, and the other side from the interface with the cast iron (Figure 1D and Figures S4A–S4C). In comparison, we have implemented the same aging, labeling, and imaging experiments on B72 coated on the quartz glass. Differently, the aging sites in B72/quartz could only be observed from the surface in contact with the air (Figure S4D). These results suggested a possible influence of cast iron on the aging evolution of B72 during the photothermal treatment, in comparison with the invalid quartz glass.

**Figure 1:**
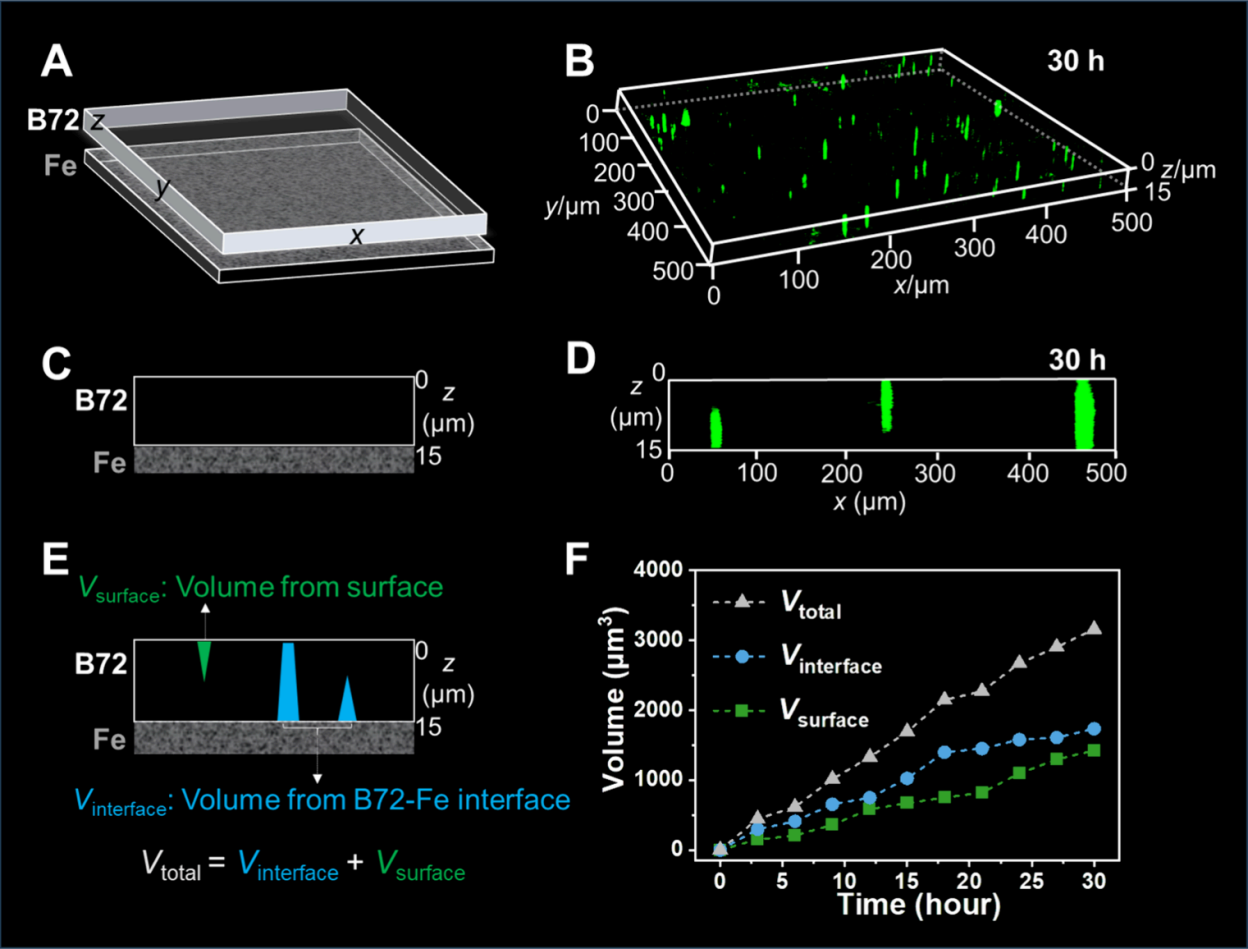
Three-dimensional fluorescence imaging and quantitative analysis of the aging process for B72 on the cast iron. (A, C, and E) Schematic representation and (B, D, and F) fluorescence imaging data for B72/Fe after photothermal treatment: (A and B) three-dimensional fluorescence images, (C and D) side-view fluorescence images, and (E and F) quantified fluorescent volumes.

We have further quantified the fluorescent volumes of the aging sites for B72/Fe during the aging (Figure 1E). The total fluorescence volume (*V*_total_) in B72/Fe increased from 0 μm^3^ at 0 h to 3159 μm^3^ after 30 h of aging treatment (Figure 1F). Interestingly, we found that the aging intrusion from different sides of B72 followed different dynamics. The volume of fluorescent sites of B72 from the interface between B72 and cast iron (*V*_interface_) reached 1735 μm^3^ at 30 h, which was larger than the volume from the surface of B72 (*V*_surface_, 1424 μm^3^ at 30 h). These results demonstrated that the cast iron could aggravate the aging behaviors of B72, which could be clearly visualized and accurately quantified through the proposed fluorescence labeling and three-dimensional imaging strategy.

### Influence of Cast Iron on the B72 Aging Evolution

In situ monitoring on the aging sites of B72 was implemented to validate the simultaneous aging evolution from both the surface and interface between cast iron and B72. It can be clearly observed that there were two kinds of fluorescent sites in the aged B72/Fe after the labeling process. Fluorescence site “i” of B72 intruded from the surface in contact with air, and it reached a depth of 14 μm after 5 h of photothermal treatment (Figure S5A). Simultaneously, fluorescence site “ii” appeared from the interface between cast iron and B72. These sites reached a depth of 13 μm at 4 h and expanded to two spots at 5 h. These visualized results provided direct evidence for the simultaneous aging evolution from the surface with air and interface between B72–iron. In comparison, a controlled sample was employed by casting B72 on the quartz glass (named as B72/quartz) under the same photothermal treatment. Similarly, the fluorescence site “i” could be visualized from the surface of B72 exposed in the air, and the depth of the aged sites intruded from 5 μm at 2 h to 14 μm at 5 h (Figure S5B). Differently, no aging evolution occurred from the interface between B72 and quartz glass. Such a difference between B72/Fe and B72/quartz demonstrated the influence of cast iron on the aging evolution of B72. Quantitatively, the total fluorescence volume of B72/Fe reached 605.5 μm^3^ after photothermal treatment of 5 h (Table S2). Notably, the fluorescence volume of site “i” of B72/Fe (258.6 μm^3^ at 5 h) grew smaller than the volume of site “ii” (346.9 μm^3^ at 5 h), suggesting a more severe aging evolution from the interface between B72 and Fe (Figure S5C). In comparison, the total fluorescence volume in B72/quartz reached 251.9 μm^3^ at 5 h, as a result of the aging from the surface (Figure S5D).

To exclude the interference of the fluorescence labeling procedures, we have conducted in situ fluorescence monitoring of B72/Fe and B72/quartz treated with different labeling time. No fluorescence signals could be observed for the unaged B72/Fe or B72/quartz under the labeling treatment from 0.5 to 2.5 h (Figures S6A–S6D). These results excluded the interference caused by the fluorescence labeling process, validating that the fluorescent sites in B72 were caused by the photothermal aging. Moreover, we have mixed iron powder (2 wt %) into B72 randomly, and the B72 film with iron powder was then photothermally treated and labeled. No fluorescent spots could be found for the unaged samples after labeling (Figures S6E and S6F). In contrast, bright fluorescent spots appeared in these iron-doped B72 samples after aging for 5 h, especially around the iron power (Figures S6G and S6H). Therefore, we conclude the influence of iron on the aging evolution of B72 through the interfacial interactions.

### Structural Changes of B72 Induced by the Cast Iron

To study the structural changes of the aged B72 on the different substrates, we have carried out gel permeation chromatography (GPC) measurements. No obvious changes could be observed for the retention time for the unaged and aged B72 (Figure 2A, inset), suggesting the absence of significant structural changes and a possible early stage aging evolution of B72.^32^ The values of weight-average molecular weight (*M*_w_) and number-average molecular weight (*M*_n_) for the B72/quartz remained stable before and after photothermal aging (Table S3). Differently, *M*_n_ for B72 on the cast iron increased from 49,897 before aging to 62,094 after aging for 30 h, while no obvious changes could be observed of *M*_w_ (Figure 2A). These phenomena could be ascribed to the simultaneous and competitive occurrence of chain-breaking and cross-linking reactions for B72 (Figure S7A) under photothermal treatment.^33^ Specifically, the chain-breaking reactions occurred in the methyl acrylate (MA) and ethyl methacrylate (EMA) segments in B72, such as the generation of vinyl α,β-unsaturated ester groups (Figure S7B, ***a***), γ-lactamide (Figure S7B, ***b***) and carboxylic acid (Figure S7B, ***c***) from MA segments and unsaturated lipids (Figure S7C, ***e*** and ***f***) from EMA segments.^34,35^ Meanwhile, cross-linking reactions occurred between the free radicals generated by the oxidation of the tertiary carbon in MA and EMA segments (Figure S7B, ***d*** and ***g***).^36,37^ Accordingly, the *M*_n_ of B72 increased due to the dominant role of cross-linking reactions in the early stage of aging.^38^ Note that the variation of *M*_n_ for B72/Fe was more prominent than that for B72/quartz, suggesting that iron may inflict an activating and catalytic effect on the aging evolution for B72.^39,40^

**Figure 2:**
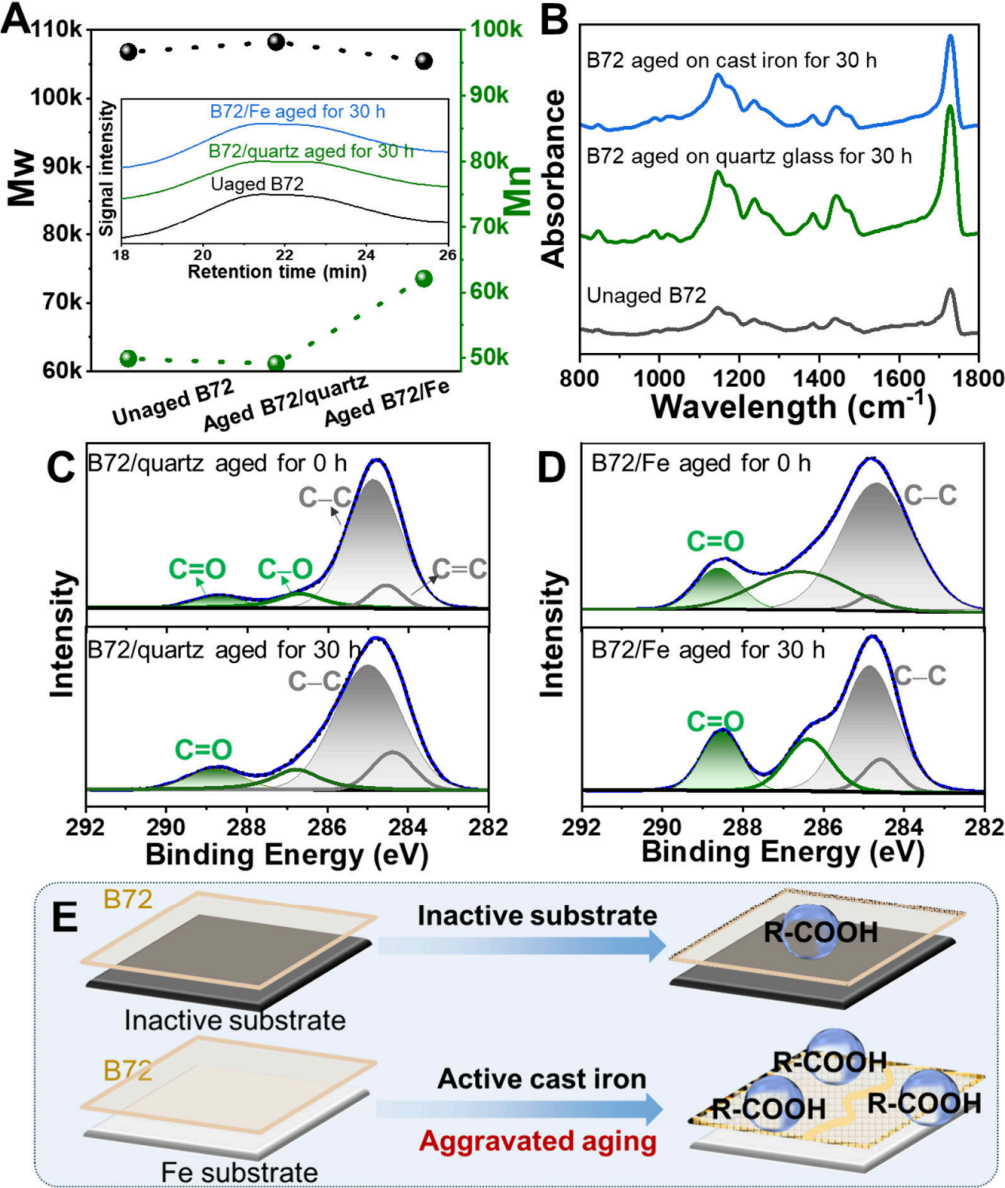
Structural changes of B72 on cast iron under photothermal treatment. (A) GPC results for the unaged B72 and aged B72 on the different substrates. (B) FT-IR data for the unaged B72 and aged B72 on the different substrates. XPS spectra of C 1s for (C) B72/quartz and (D) B72/Fe before and after photothermal treatment for 30 h. (E) Schematic representation for the effect of inactive and active substrates on the aging evolution for B72.

Fourier transform infrared (FT-IR) spectroscopy was implemented to study the changes of the unaged B72, B72 aged on cast iron, and B72 aged on quartz glass. Typical characteristic bands ascribed to the C–O–C in the ester group could be observed at 1240, 1140, and 1020 cm^–1^ for these B72 samples (Figure 2B). The band at 1729 cm^–1^ was attributed to the stretching vibration of C=O, and the bands at 1440 and 1385 cm^–1^ were ascribed to the C–H bonds.^41,42^ To quantify the aging evolution for these B72 samples, the carbonyl index $\left(CI=\frac{A_{1729}}{A_{1440}}\right)$ was calculated.^43^ The CI value of the unaged B72 was 2.85, while the value increased to 3.02 and 4.23 for the B72/quartz and B72/Fe, respectively (Table S4). Obviously, the structural changes of B72 on cast iron (ΔCI = 1.38) were much larger than that of B72 on the quartz glass (ΔCI = 0.17) under the same aging conditions, indicating the active role of cast iron to induce and accelerate the aging of B72.

X-ray photoelectron spectroscopy (XPS) was further carried out for validation. The C 1s spectra of the unaged B72/quartz could be deconvoluted into C=C (284.6 eV), C–C (284.9 eV), C–O (286.7 eV), and C=O (288.7 eV),^44,45^ and the oxidation status of the samples could be evaluated by calculating the area ratio of C=O to C–C.^46^ It could be observed that this ratio changed from 0.08 to 0.13, with the increment of 0.05, for B72/quartz after photothermal treatment for 30 h (Figure 2C and Table S5). In comparison, the increment for this ratio was promoted to 0.11 after the same treatment for B72/Fe (Figure 2D), indicating a more intense aging behavior of B72/Fe than that of B72/quartz. Based on these results, we can conclude that the active iron substrate could accelerate the generation of carboxyl groups and thus aggravated the aging evolution of B72, while the inactive substrate showed no influence on the aging evolution of B72 (Figure 2E). These phenomena could be ascribed to the ability of iron ions to capture the electrons from the B72 with negatively charged carboxyl groups, leading to the oxidation reaction for chain breaking and cross-linking for B72.^47^

### Structural Changes of Cast Iron Induced by the Aged B72

It is inferred that the aged B72 would inflict influence on the structures of the cast iron. First, electron spin resonance (ESR) spectroscopy was implemented to study the possible reactive oxygen species that generated during the aging process of B72. Self-supported B72 was prepared and treated under photothermal for 50 h. Obvious signals ascribed to the hydroxyl radicals (^•^OH) could be observed for the aged B72, while no signal was observed for B72 before treatment (Figure 3A). These results showed the formation of ^•^OH during the photothermal aging of B72, which may be harmful to the cast iron or metal artifacts.^48,49^ Second, the generated carboxyl groups in the B72, as observed from fluorescence images, would result in the formation of carboxylic acid with acidic environment. The pH values of the leaching solution from self-supported B72 after aging treatment were tested. The results showed that the pH values decreased from 6.9 before aging to 6.2 after aging for 5 h, followed by a continuous decrease to 60 h (Figure 3B). The increased acidity could also be validated from a planar pH meter recording the decreased pH values of the self-supported B72 during aging treatment for 60 h (Figure S8A). These acidic substances generated during the aging evolution of B72 threaten to induce the corrosion of metal, resulting in the consequent damage of the cast iron.^50^ For validation, a UV–vis absorption spectrum of iron powder in the presence of long-chain acid, lauric acid as an example, was recorded to study the variation of iron. The absorbance around 245 nm could be observed (Figure S8B), indicating that iron ions were produced from iron powder under acidic conditions.^51^ These results suggested the possibility that the acidic environment provided by the aged B72 would induce the variations of valence state for cast iron during the accelerated photothermal aging process.

**Figure 3:**
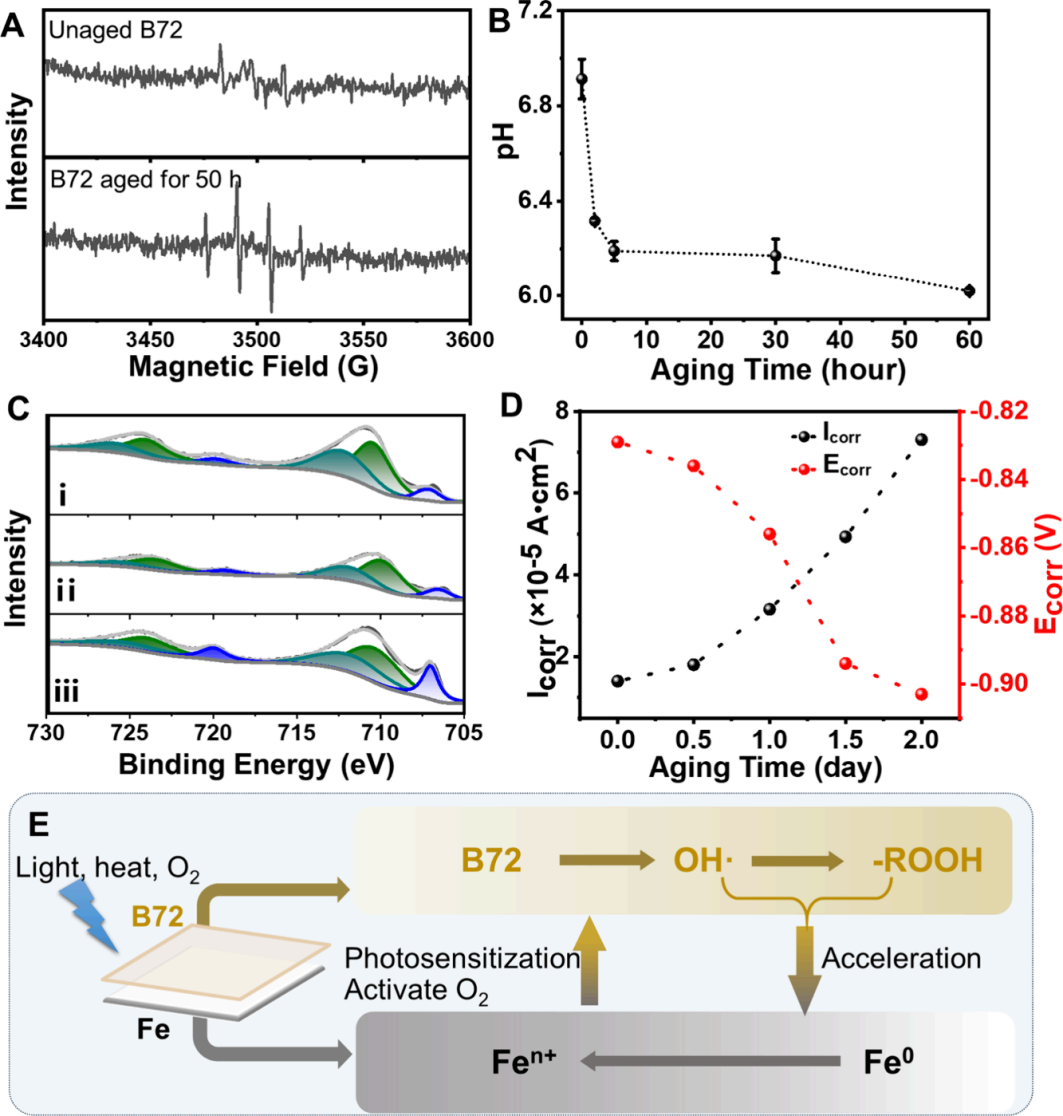
Influence of aged B72 on the cast iron. (A) ESR spectra of unaged B72 and B72 aged for 50 h. (B) pH measurements of the leaching solution for the aged self-supported B72 (the error bar represented the standard deviation from three parallel tests). (C) XPS spectra of Fe 2p on the surface of cast iron: (i) B72/Fe aged for 30 h and removed B72, (ii) bare cast iron aged for 30 h, and (iii) bare cast iron aged for 0 h (blue lines for Fe(0), green lines for Fe­(II), and olive lines for Fe­(III)). (D) Changes of corrosion potential (*E*_corr_) and current densities (*I*_corr_) of cast iron coated with B72 after photothermal aging. (E) Schematic representation for the interaction between B72 and cast iron during the photothermal treatment.

To confirm the changed valence state of the cast iron, we have measured the XPS spectra of Fe 2p for the cast iron. It can be observed that the binding energies of Fe are located around ∼707, ∼710, and ∼712 eV, indexing to the Fe(0), Fe­(II), and Fe­(III), respectively (Table S6). Obvious peaks for Fe(0) could be observed for the bare cast iron without aging treatment (Figure 3C, blue lines), ascribing to the stable existence of iron in the cast iron. Upon photothermal treatment, the peaks attributed to Fe­(II) and Fe­(III) grew significantly in the bare cast iron (Figure 3C, green and olive lines), along with the decayed peaks of Fe(0). Quantitative analysis was implemented by calculating the area ratio of the [(Fe­(III) + Fe­(II))/Fe(0)] to study the contents of the iron ions. This ratio for the bare cast iron increased from 4.21 before photothermal treatment to 7.82 after treatment for 30 h, suggesting that possible oxidation occurred for the cast iron without the coating of B72. In comparison, we coated B72 on the cast iron and conducted the same aging treatment. The XPS measurements and valence state calculation of Fe in B72/Fe were implemented after removing the B72. No obvious changes could be observed for the unaged B72/Fe after removing the B72 (Figure S8C), indicating that the coating and removing process would not induce a possible reaction for the cast iron. Surprisingly, the area ratio of [(Fe­(III) + Fe­(II))/Fe(0)] for B72/Fe increased significantly to 11.98 after photothermal treatment for 30 h. Such a large increase demonstrated that abundant iron ions were formed in the cast iron due to the oxidation reaction in the presence of the aged B72. These results indicated that the aged B72 would induce severe structural changes of cast iron.

This finding has arisen the query for the protective ability of B72 on the cast iron. We have further implemented the electrochemical studies to figure out the variations of cast iron in the presence or absence of the B72 coatings. The corrosion current densities (*I*_corr_) and corrosion potential (*E*_corr_) of the iron were calculated by the Tafel extrapolation method based on their polarization curves. It could be observed that the values of *I*_corr_ and *E*_corr_ of bare cast iron before aging treatment were about 5.16 × 10^–4^ A/cm^2^ and −0.909 V, respectively, and these two values remained almost stable after photothermal treatment for 2 days (Tables S7 and S8). Coating of B72 has effectively inhibited the corrosion of iron, and the values of *I*_corr_ and *E*_corr_ for the unaged B72/Fe changed to 1.40 × 10^–5^ A/cm^2^ and −0.829 V (Figures S8D and S8E). Upon the photothermal treatment for 2 days, the *I*_corr_ value of B72/Fe increased to 7.31 × 10^–5^ A/cm^2^ and the *E*_corr_ decreased to −0.903 V (Figure 3D). Due to the positive correlation between corrosion rate and current density, the larger current density corresponded to the higher corrosion rate of iron.^52^ The obvious decrease of corrosion potential for B72/Fe indicated that the cast iron with coatings was more prone to corrosion.^53^ Accordingly, it can be concluded that the aging process of B72 contributed to the corrosion of cast iron.

Based on the above findings, we can conclude that the strong interaction existed at the interface between B72 and iron. The methyl acrylate (MA) units in B72 are apt to age under the photothermal treatment,^29^ generating hydroxyl radicals (^•^OH) and broken chains ending with carboxyl groups. With the proceeding aging evolution, the aging sites of B72 developed at both the surface and the interface with Fe. Due to the ability of ferrous ions to activate oxygen,^40^ the ferrous ions in the cast iron exhibited a photosensitization effect to aggravate the aging evolution of B72 at the B72/Fe interface. Some penetrable aging sites were found, acting as channels to transport oxygen and water to the B72/Fe interfaces.^34^ Accordingly, a weak acidic microenvironment was formed at the B72/Fe interface, triggering the oxidation of iron to the ferrous ions (Figure S9). Moreover, the iron could also be oxidized to the ferrous ions in the presence of the oxidant ^•^OH.^48,54^ Therefore, we can deduce that reactive oxygen species (^•^OH) and acidic carboxyl groups were generated during the aging evolution of B72, leading to the oxidation and corrosion of iron to generate iron ions. Simultaneously, these iron ions would photosensitize the aging evolution of B72.^55^ Therefore, an influence cycle was formed, resulting in the aggravated aging evolution of B72 and accelerated corrosion of cast iron (Figure 3E).

### Coating Effect of B72 on Metal Artifacts

To verify the practicability of our proposed method in metal artifacts, we have selected iron debris from the Nanhai No. 1 and iron coin from the Northern Song for monitoring. We have noticed that there was plentiful iron rust on these metal artifacts, and thus we have first studied the effect of the iron rust on the aging evolution of B72 through the in situ monitoring. As expected, aging sites appeared from both the surface of B72 and the interface between B72 and iron rust. The aging sites from the surface of B72 appeared at 3 h and intruded to 18 μm at 5 h, and the depths of the aging sites from the B72–rust interface increased to 16 μm after 5 h of treatment (Figure S10A). Note that the volume from both the interface and the surface expanded aggressively (Figure S10B). The total volumes of the aging sites varied according to the following tendency: B72/rust (725 μm^3^) > B72/Fe (605 μm^3^) > B72/quartz (251 μm^3^, Figure S10C). This result suggested that the iron rust with abundant active iron would induce much more severe aging of B72 than the cast iron or quartz glass, validating the demonstration that the oxidized iron would intensify the aging evolution of polymer coatings.

B72 was coated on the iron debris from the Nanhai No. 1, and the treatment and aging evaluation of B72 was implemented as follows. First, the nodulated rust on the iron debris was mechanically derusted according to a typical surface clearing procedure,^56^ and a black dense rust layer was exposed on the surface (Figure 4A, step (i) to (ii)). Second, the treated iron debris was coated with B72, and the thickness of B72 coatings from different positions on the debris remained almost consistent with the thickness of approximately 12 μm (Table S9). Afterward, the B72-coated debris was aged under photothermal treatment, and the fluorescence labeling and imaging was implemented in the region framed by yellow dotted lines (Figure 4A, step (iii)). Abundant fluorescence sites with the total volume of 1555 μm^3^ were observed in the three-dimensional images for the B72 on the iron debris after aging for 30 h (Figure 4B), while a clear background could be observed for this B72 without photothermal treatment or labeling procedures (Figure S11A). These fluorescence sites appeared from both the surface of B72 and the interface with the iron debris, which could be identified by the side-view images from the in situ monitoring during the treatment of 5 h (Figure 4C). The intruded fluorescence sites demonstrated the formation of carboxyl groups from B72 during the photothermal treatment. Similar results could also be acquired from the iron coin acquired from the Northern Song. The three-dimensional fluorescence images showed that the aging evolution of B72 appeared from both the surface in the air and the interface between iron coin and B72 (Figures S11B–S11D). Moreover, it is delightful that the aging sites of B72 on a slightly inclined section in the artifacts could also be observed in the three-dimensional fluorescence images (Figure S11E). Therefore, the proposed strategy could be applied in metal artifacts. We can draw a conclusion that the aging of B72 occurred or even aggravated at the interface contacted with metal artifacts, leading to the generation of carboxyl groups and active oxygen species.

**Figure 4:**
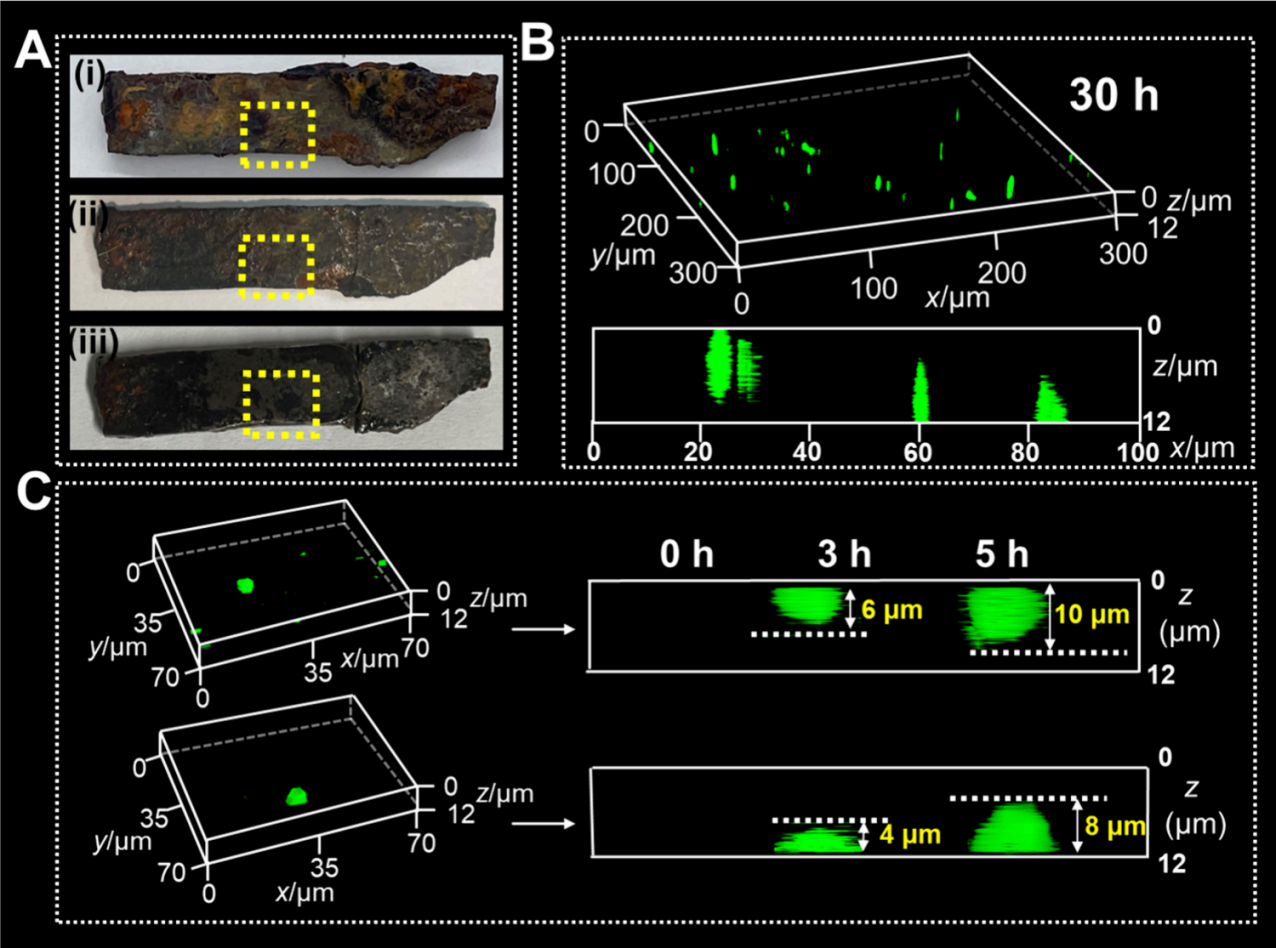
Three-dimensional fluorescence analysis of B72 on the iron debris from Nanhai No. 1. (A) Photos of iron debris from Nanhai No. 1 (i, untreated; ii, rust removed; iii, coated with B72 and fluorescent labeled). (B) Three-dimensional and side-view fluorescence images of iron debris from Nanhai No. 1 coated with B72 and photothermally treated for 30 h. (C) In situ monitoring of the aging sites of B72 on iron debris from Nanhai No. 1.

A micro-Raman technique was further implemented to analyze the varied composition of rust species on the surface of metal artifacts from Nanhai No. 1 (Figures S12A and S12B). For the unaged iron debris coated with B72, most Fe existed as stable phases of Fe_3_O_4_ and α-FeOOH, and a small number of unstable phases β-FeOOH could be identified from the Raman spectra (Figure 5A). After photothermal treatment for 90 h, unstable corrosion products β-FeOOH and γ-FeOOH appeared along with Fe_3_O_4_ and α-FeOOH phases (Figure 5B). Semiquantitative component analysis from Raman scanning mappings were implemented for different rust products (Figures 5C and 5D and Table S10), and the protection ability index (PAI, (Fe_3_O_4_ + α-FeOOH)/(β-FeOOH + γ-FeOOH)) was calculated.^57^ The value of PAI decreased from 4.28 at 0 h to 3.46 at 90 h. The decayed PAI suggested the increased corrosion rate of iron rust after photothermal treatment, as a result of the deteriorated protective ability of B72.

**Figure 5:**
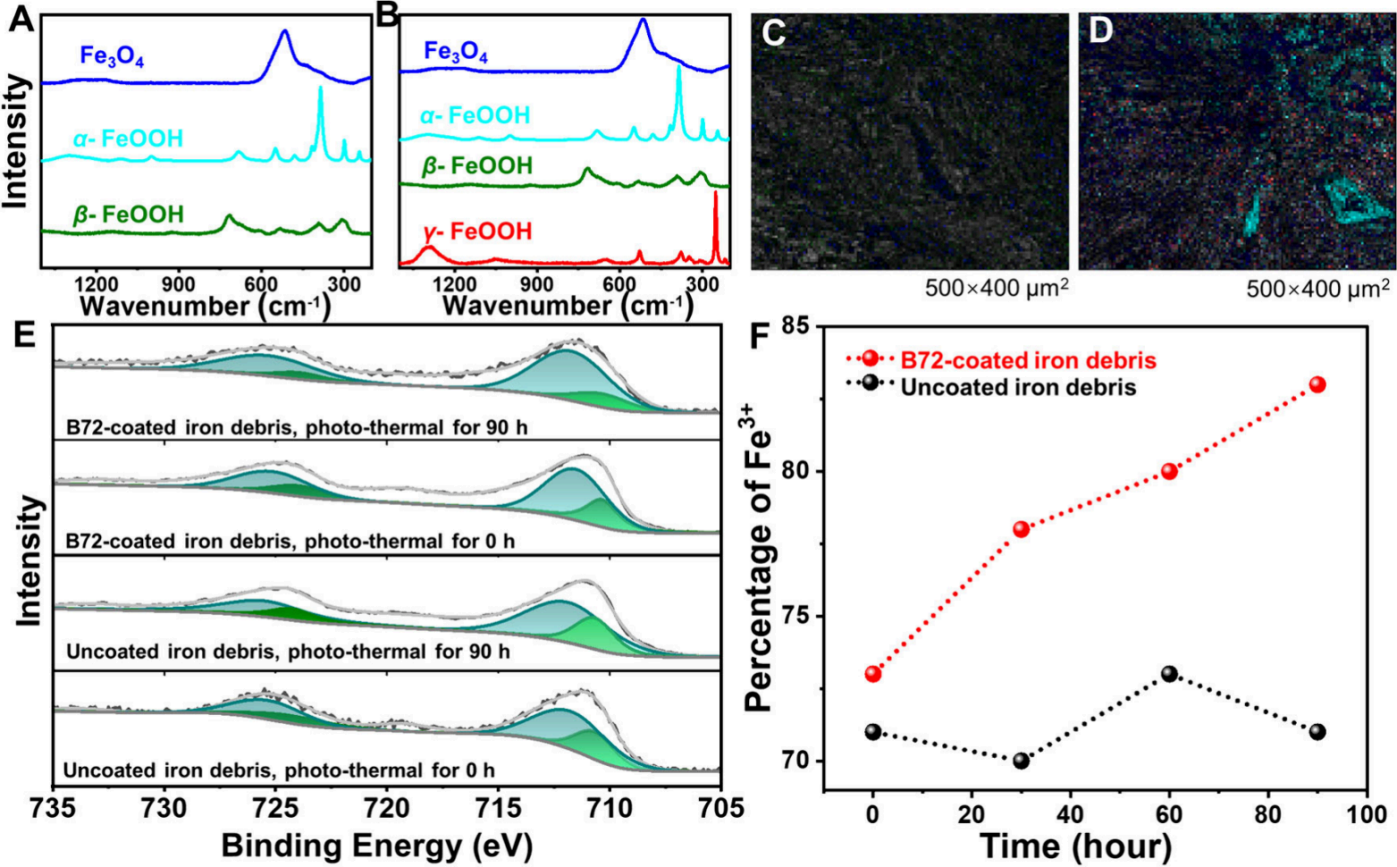
Structural changes of iron debris from Nanhai No. 1 induced by the aged B72. (A and B) Raman spectra and (C and D) scanning mappings of the surface for iron debris from Nanhai No. 1 (A and C) before and (B and D) after photothermal treatment for 90 h (blue for Fe_3_O_4_, cyan for α-FeOOH, green for β-FeOOH, and red for *γ-*FeOOH). (E) XPS spectra of Fe 2p (green lines for Fe­(II) and olive lines for Fe­(III)). (F) Varied contents of Fe^3+^ for iron debris from Nanhai No. 1 before and after photothermal treatment for different time. All the coated B72 was removed before the Raman and XPS measurements.

Furthermore, XPS spectra of iron rust on the iron debris from Nanhai No. 1 were analyzed during the photothermal treatment in the absence or presence of B72 coatings. Both Fe­(II) and Fe­(III) could be identified in these samples at the binding energies around 710 and 711 eV (Tables S11 and S12). For the iron species in the absence of B72, the proportions of Fe­(II) and Fe­(III) remained stable during the photothermal treatment for 90 h (Figure 5E and Figure S12C), suggesting relatively stable phase compositions during the treatment. In contrast, the compositions of iron species varied distinctly for the iron debris coated with B72. The contents of Fe­(III) increased gradually from 73% to 83% during the photothermal treatment of 90 h (Figure 5F). This variation could be ascribed to the transition from the stable Fe_3_O_4_ to the active and harmful γ-FeOOH.^1,58^ These results clearly pointed out that the aged B72 would show the chemical damages to the metal artifacts during the photothermal treatment. Therefore, thoughtful considerations should be taken on the polymer coatings in order to evaluate both the lifetime and safety of the polymer coatings and provide better protection for cultural artifacts.

## Conclusions

In summary, we have proposed an in situ three-dimensional strategy to visualize the early stage aging behaviors of the polymer coatings on the metal artifacts, especially at the interfaces between polymers and artifacts. It is surprising to acknowledge that the aged polymers can generate hazardous carboxyl groups and reactive hydroxyl radicals, inducing the oxidation and corrosion of the metal artifacts. On the other hand, the oxidized metallic ions would activate the chain scission and aggravate the aging of B72. These findings are significant and alarming for the artifact conservation. In view of these facts, some suggestions are put forward for the polymer coatings on the artifact conservation. First, the storage environment of artifacts should be strictly controlled, in the presence or absence of polymer coatings. Second, we should strengthen the research on the modification of polymer coatings, including the structural design and preparation optimization, to exclude the existence of defects or pores in the polymers. Employment of a specific stabilizers or anti-aging additives into polymer coatings is also significantly needed. These actions would promote the protective ability of polymer coatings and reduce the secondary damage to cultural artifacts. We will keep up the corresponding research on the preservation of cultural artifacts, and it is anticipated that the proposed strategy and findings could provide sufficient information for artifact conservation.

## Supplementary Material



## Notes

Materials, measurements, experimental section, potentiometric curve, three-dimensional fluorescence images, volume changes from the imaging, pH curves, UV–vis absorption spectra, XPS spectra, polarization curves, reaction mechanism study, and Raman microscopic imaging of the samples (PDF)
