# Supplementary material for: Unexpected Damage on Metal Artifacts Triggered by the Hazardous Interfacial Interaction from Aging of Polymer Coatings
Source: ACS Cent Sci. 2025 Apr 23;11(5):694–703. doi: 10.1021/acscentsci.5c00067 (PMC12123457; doi:10.1021/acscentsci.5c00067)
Supplement: Supplementary file 1 [file oc5c00067_si_001.pdf]

Supporting Information for

Unexpected Damage on Metal Artifacts Triggered by the Hazardous Interfacial Interaction from Aging of Polymer Coatings

Ying An,[a] Pei Hu,[a] Kaitao Li,[a,c] Yu Kang,[a] Gang Hu,[d] Rui Tian,*[a,c] Chao Lu*[a,b,c], and Xue Duan[a,c]

[a]*State Key Laboratory of Chemical Resource Engineering, Beijing University of Chemical Technology, Beijing, 100029, China*

[b]*Pingyuan Laboratory, College of Chemistry, Zhengzhou University, Zhengzhou, 450001, China*

[c]*Quzhou Institute for Innovation in Resource Chemical Engineering, Quzhou, 324000, China*

[d]*School of Archaeology and Museology, Peking University, Beijing, 100871, China*

*Fax/Tel.. +86 10 64411957. E-mail. tianrui@mail.buct.edu.cn; luchao@mail.buct.edu.cn.

Materials

All the reagents in the experiments were of analytical grade and used without further purification. Acetone was purchased from Sinopharm Chemical Reagent Co., Ltd. Paraloid B72 (copolymer of ethyl methacrylate and methyl acrylate), sodium polymethacrylate (PMAA), 6-aminofluorescein (AF), 1-(3-dimethylaminopropyl)-3-ethyl carbodiimide hydrochloride (EDC), and N-hydroxysuccinimide (NHS) were purchased from HWRK Chem Co., Ltd. (Beijing, China). Dimethyl sulfoxide (DMSO) was obtained from Beijing Chemical Reagent Company. Potassium chloride (KCl) and disodium hydrogen phosphate (Na_2HPO_4) were acquired from Beijing Reagent Company (Beijing, China). Sodium dihydrogen phosphate (NaH_2PO_4) was purchased from Tianjin Jinke Fine Chemical Research Institute (Tianjin, China). Sodium sulfate was bought from Fuchen Chemical Reagent Co., Ltd. (Tianjin, China). 5,5-Dimethyl-1-pyrroline N-oxide (DMPO) was acquired from J&K Scientific Co., Ltd. (Beijing, China). The gray cast iron (10 mm × 10 mm × 1 mm) was brought from Hefei Wenghe Metal Materials Co., Ltd.. The content of Fe in the gray cast iron was determined as 93.74%, and the contents of C, Si, Mn, S and P were 3.21%, 1.82%, 0.98, 0.097 and 0.15%, respectively. The real artifacts were acquired from the iron fragments salvaged from the Nanhai No. 1 shipwreck and the iron money from the Northern Song Dynasty.

Measurements

The fluorescence changes of the B72 films were captured using a Leica TCS SP8 confocal laser scanning microscope (CLSM) with quantitative data acquired from Leica Application Suite X. The B72 molecules in the B72/Fe and B72/quartz samples were dissolved by acetone, and then the solution was dropped on the potassium bromide tablet to record the Fourier transform infrared spectrum using a Nicolet 6700 (Thermo Electron) instrument in the range of 400 – 4000 cm^{-1}. X-ray photoelectron spectroscopy (XPS) of the samples was carried out on ESCALAB 250 (Thermo Fisher Scientific, USA) using an monochromated Al Kalph 150 W source. Electrochemical signals of the aged B72/Fe were measured by the CHI 660E electrochemical workstation (Chenhua Instrument, China). Electron spin

resonance (ESR) spectra of the aged B72 films were recorded on a X-band Bruker E500 spectrometer (Bruker, Germany). The gel permeation chromatography (GPC) tests for the aged B72 were performed using the Waters 1515 pad gel permeation chromatograph with tetrahydrofuran (THF) as the solvent. The pH of the leaching solution for the aged B72 was determined using a bench pH meter model FE28, branded METTler Toledo. The Raman spectra of the sample were collected on the Renishaw inVia Raman spectrometer (United Kingdom), and the selected area was scanned using the Map image acquisition mode. The samples were monitored under Lecia microscope 100× conventional objective. The wavelength of the laser was 532 nm, the scanning area was 400 × 500 μm^2, the step size was 3 μm, and the laser power was 1% (0.15 mW). The spectral fitting method was used for semi-quantitative analysis of Raman data, and nonnegative least squares (NNLS) was used to analyze Raman data to obtain semi-quantitative results of the relevant components.

Experimental Section

Coating treatment of the cast iron by B72. Before the coating process, the surface of grey cast iron was cleaned by the detergent and polished by the sandpaper of 600, 1500 and 8,000 particle size, respectively. The polished cast iron was coated by B72 in acetone (weight content of 10%), and the casting process was implemented by a homogenizer with the rotating speed of 3300 r/min for 6 seconds. The acquired samples were labelled as "B72/Fe".

Similarly, the iron fragments from the cultural artifacts were placed in the oven (120 °C) for 4 hours, and the surface of these artifacts was mechanically treated with sandpaper with particle size of 600, 1500 and 8000, respectively. After washing in acetone, the metal artifacts were coated with B72 (weight content of 10%) by brushing, and the acquired samples were labelled as "B72/artifact".

Photo-thermal treatment of the B72-coated cast iron. Photo-thermal treatment of the B72-coated cast iron was implemented in a Q-Lab QUV UV/spray accelerated weathering equipment. The temperature was set at 60 ºC, and the UV irradiation condition was 1.0 W/m^2 with the wavelength of 340 nm. The aged cast iron was sampled every 3 hours.

Fluorescent labelling and in-situ monitoring. The AF was first dissolved in DMSO at a concentration of 10 mmol/L. This AF stock solution was then diluted to 0.12 mmol/L by EDC (1.0 mmol/L), NHS (1.0 mmol/L) and buffer solution with pH of 5.0. Afterwards, the B72/Fe and B72/artifact samples were immersed in AF solution (0.12 mmol/L) for 30 minutes to achieve a fully fluorescent labelling. Then, the samples were rinsed with deionized water (1 min) to remove the free AF molecules. Finally, the B72 samples were naturally dried and fixed onto the sample table for fluorescence imaging.

To capture the fluorescence changes of the dyed B72 on the cast iron or the cultural artifacts, the laser at 488 nm was used as the excitation light, and the fluorescence emission was collected in the range of 515 – 565 nm. The lengths of the X-axis and Y-axis for the captured images were 1.16 mm, and the depth of the Z-axis was set at 15 μm with the step of 0.1 μm.

Controlled experiments of B72 on the different substrates. As references, B72 was also coated on the quartz glass through the spinning coating method. The quartz glass (20 mm × 20 mm × 1 mm) was cleaned by detergent, followed by the ultrasonic treatment in acetone for 2 min. The B72 was coated on the quartz glass by the homogenizer with the speed of 3300 r/min for 6 seconds. The acquired samples were labelled as "B72/quartz". The aging treatment, fluorescent labelling, and imaging processes of the B72/quartz were implemented in the similar procedures as those on the cast iron.

Moreover, self-supported B72 films were prepared by the solvent evaporation strategy. Briefly, 15 mL of B72 acetone solution with a mass fraction of 10% was placed in a dish with a diameter of 20 cm and left at room temperature for 5 hours to volatilize the acetone. The acquired films were peeled off gently and labelled as "self-supported B72". The conditions for the photo-thermal aging were in accordance with the B72 on the other substrates.

In addition, we have mixed iron powder into B72 at a weight fraction of 2%. The iron powder was dispersed in B72 (weight content of 10% in acetone) randomly, and the acquired solution was casted on quartz glass by a homogenizer with the rotating speed of 3300 r/min for 6 seconds.

Polarization curve measurements. The polarization curves of cast iron samples coated with B72 after photothermal treatment were measured by CHI 660E electrochemical workstation. The B72/Fe sample

was used as the working electrode, and the saturated calomel electrode and Pt electrode were employed as the reference electrode and auxiliary electrode, respectively. Aqueous solution of 2 wt% Na_2SO_4 (pH = 7.0) was used as the experimental electrolyte.

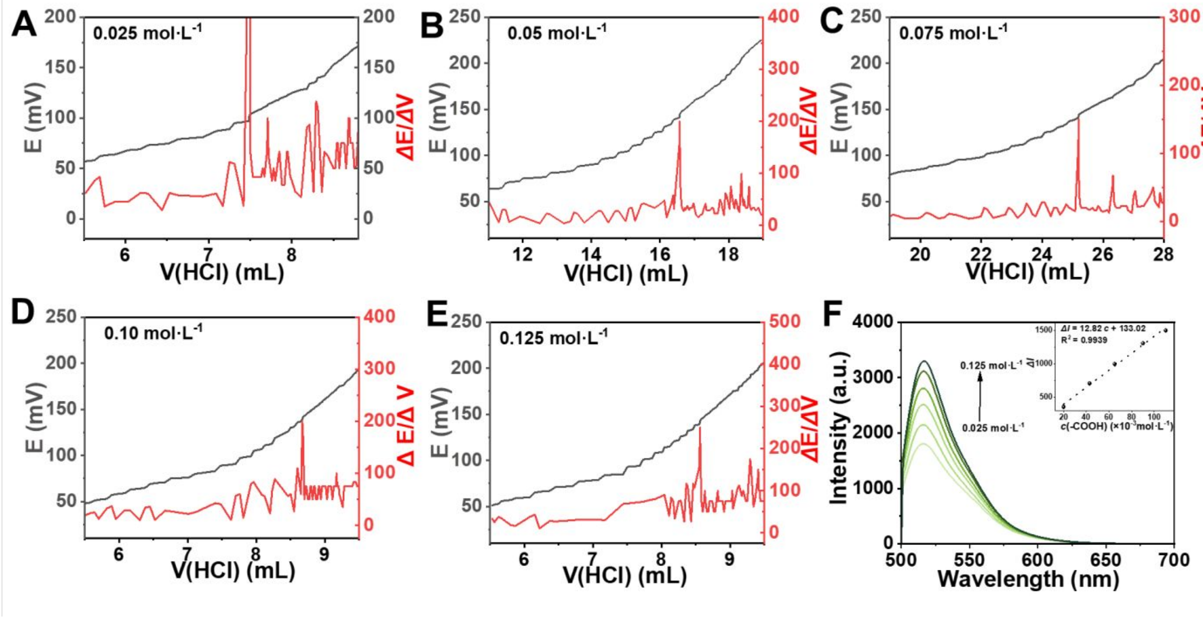

Figure S1. Quantitative analysis of carboxyl groups. Titration curves for sodium polymethacrylate (PMAA) at different theoretical concentrations. (A) 0.025 mol/L, (B) 0.05 mol/L, (C) 0.075 mol/L, (D) 0.10 mol/L and (E) 0.125 mol/L. (F) Fluorescence intensity changes of 6-aminofluorescein (AF) (0.12 mmol/L) in the presence of PMAA with different concentrations of carboxyl groups, and the inset showed the fitting equation (λ_{ex} = 490 nm).

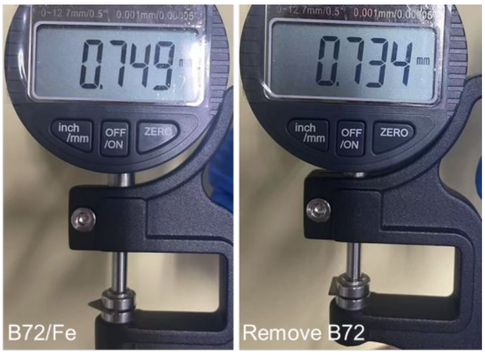

Figure S2. Measurements of the thickness of B72 coatings on the cast iron. (A) thickness of B72/Fe and (B) thickness of bare cast iron after removing B72.

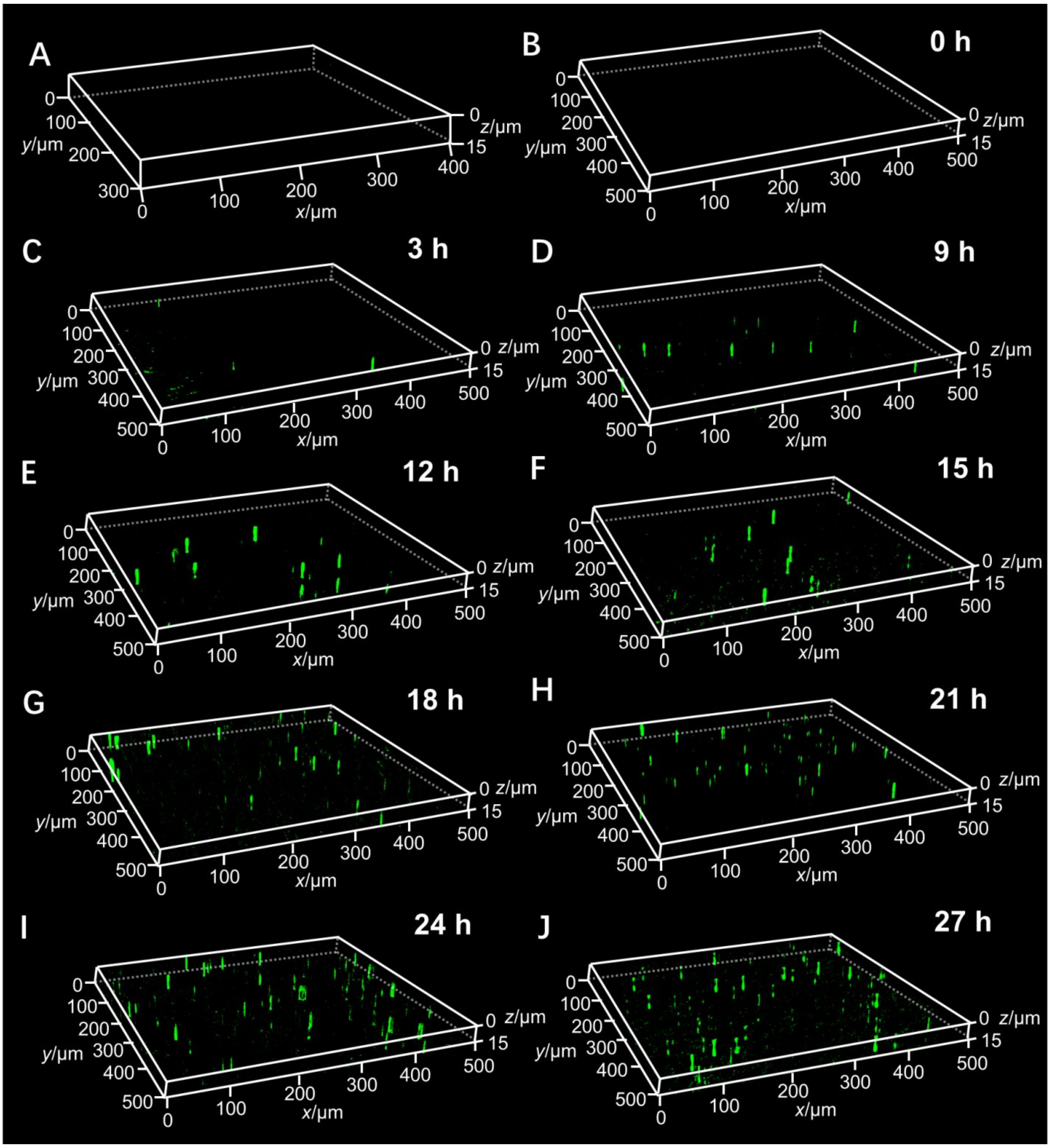

Figure S3. Three-dimensional fluorescence imaging of B72/Fe after different time of photo-thermal aging. (A) Aging for 30 h without fluorescent labeling (λ_{ex} = 488 nm). (B–J) Aging for different time after fluorescent labeling, and the aging time changed from (B) 0 h, (C) 3 h, (D) 9 h, (E) 12 h, (F) 15 h, (G) 18 h, (H) 21 h, (I) 24 h to (J) 27 h.

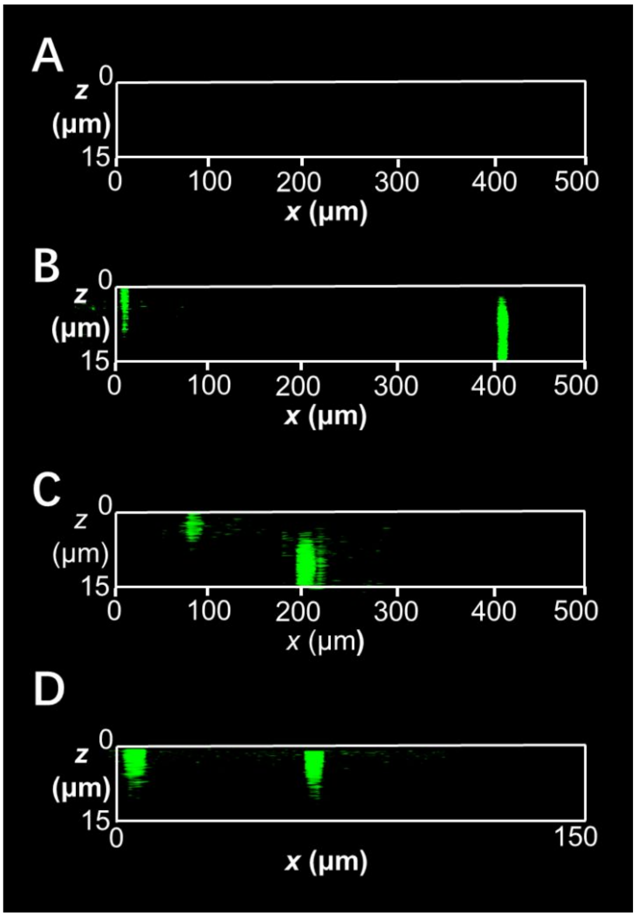

Figure S4. Side-view fluorescence imaging of B72/Fe and B72/ quartz. B72/Fe aged under photo-thermal for (A) 0 h, (B) 12 h and (C) 21 h. (D) B72/quartz under photo-thermal aging for 6 h.

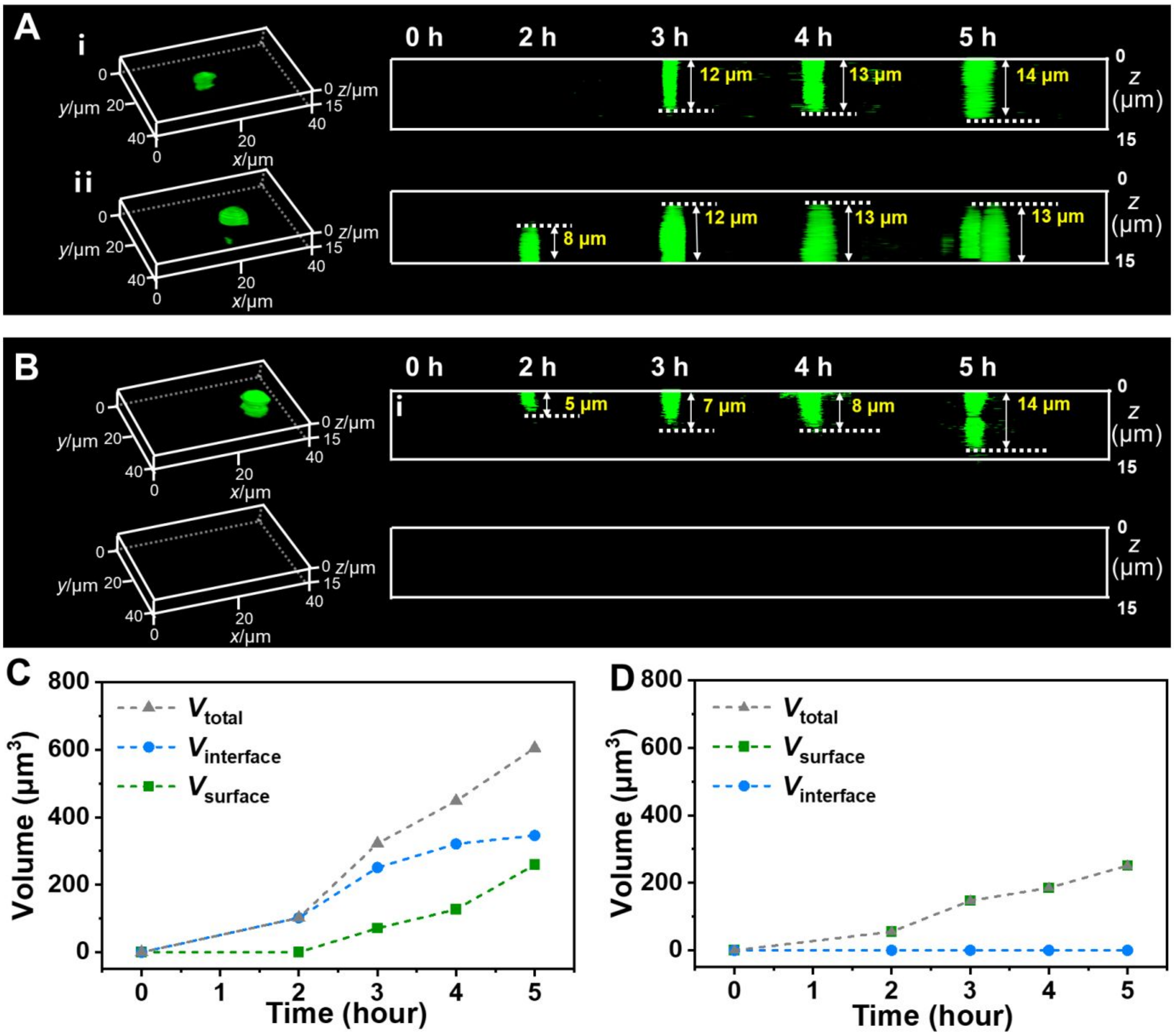

Figure S5. In-situ three-dimensional fluorescence analysis of B72 on the cast iron and quartz glass. (A, B) In-situ three-dimensional fluorescence images and (C, D) variations of the fluorescence volumes for the in-situ monitoring of (A, C) B72/Fe and (B, D) B72/quartz under photo-thermal treatment.

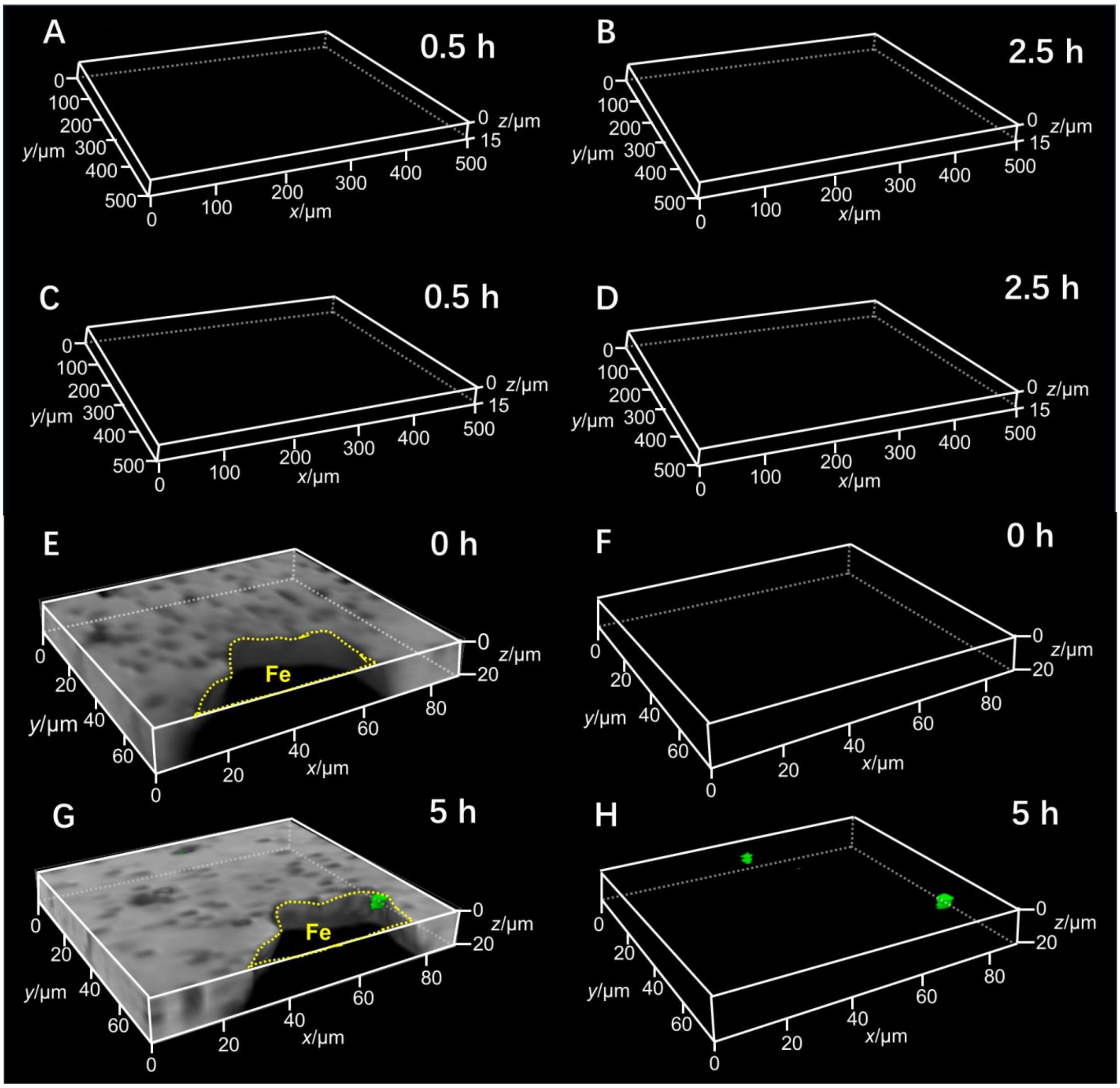

Figure S6. Study on the influencing factors for the fluorescence imaging during the labelling and imaging process. In-situ three-dimensional fluorescence images of (A, B) unaged B72/Fe and (C, D) unaged B72/quartz after labelling for (A, C) 0.5 h and (B, D) 2.5 h. (E, G**)** Merged and (F, H) fluorescence images of B72 doped with Fe powder (E, F) before and (G, H) after photo-thermal aging for 5 h, and the yellow dash circled the location of Fe powder in the B72 film.

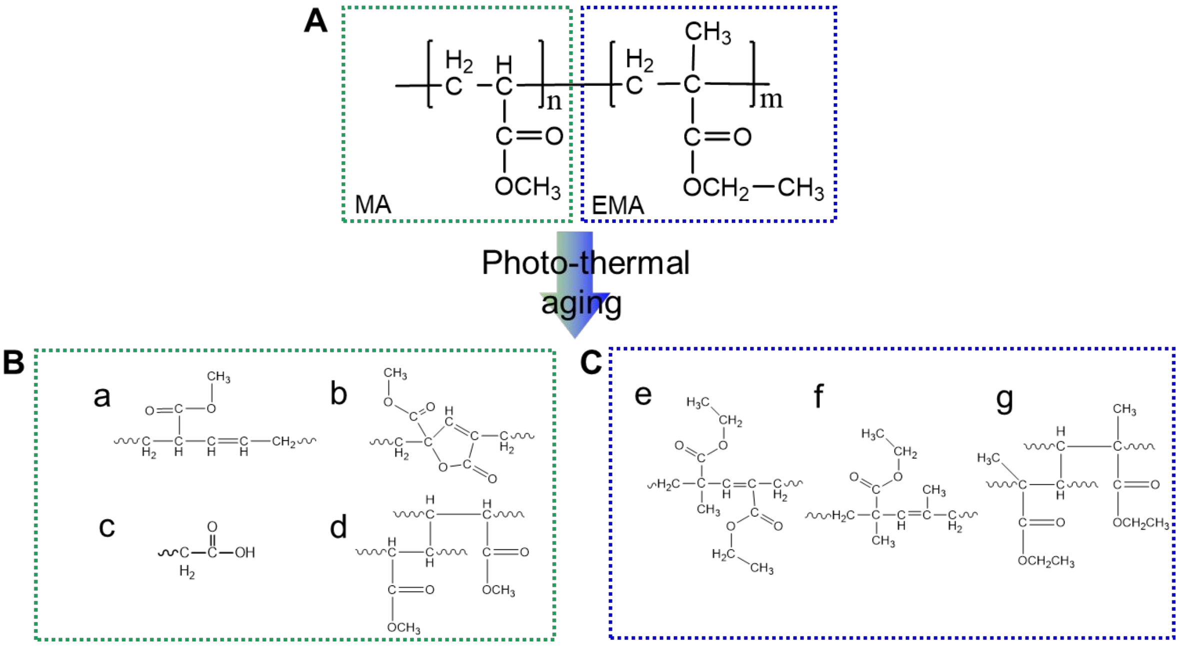

Figure S7. Schematic representaion for the reactions of B72. (A) Chemical structural formula of B72; aging products of (B) MA and (C) EMA segments.

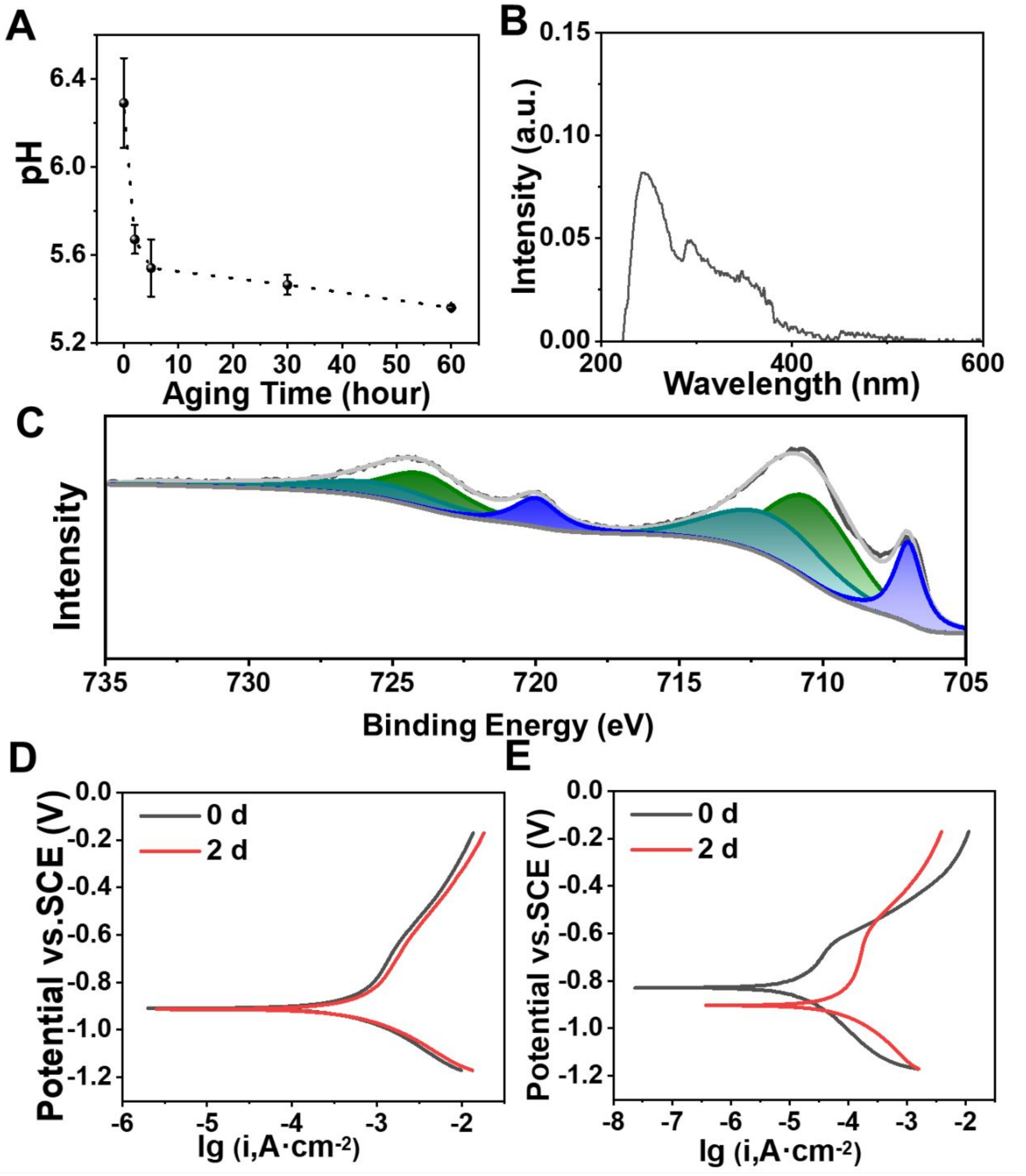

Figure S8. Influence of aged B72 on cast iron under photo-thermal treatment. (A) pH variations of self-supported B72 after photo-thermal aging for different time, and the error bar represented the standard deviation of three parallel tests of the same device. (B) UV-vis absorption spectrum of Fe powder after reaction in lauric acid solution. (C) XPS results of Fe 2*p* on the bare cast iron without aging treatment. Polarization curves of (D) bare cast iron and (E) cast iron coated with B72 before and after photo-thermal treatment for 2 days.

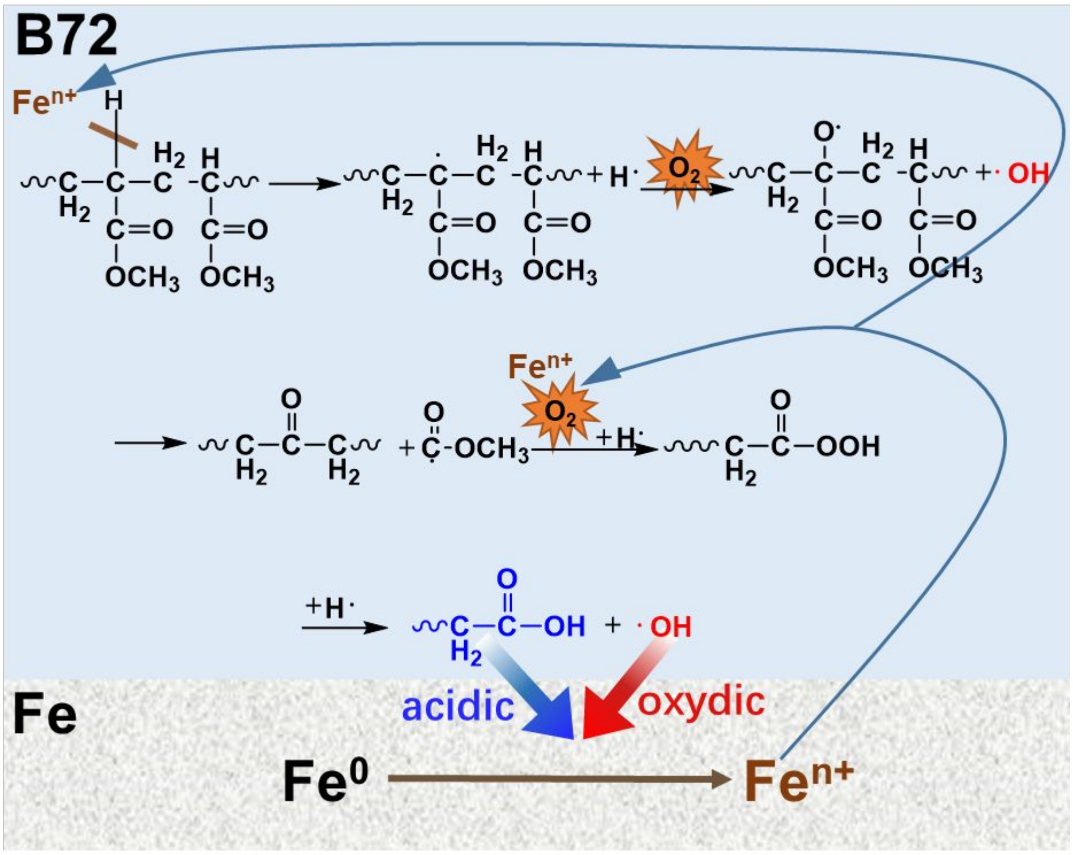

Figure S9. Reaction mechanism study. Interaction between B72 and cast iron at B72/Fe interface under photo-thermal aging treatment.

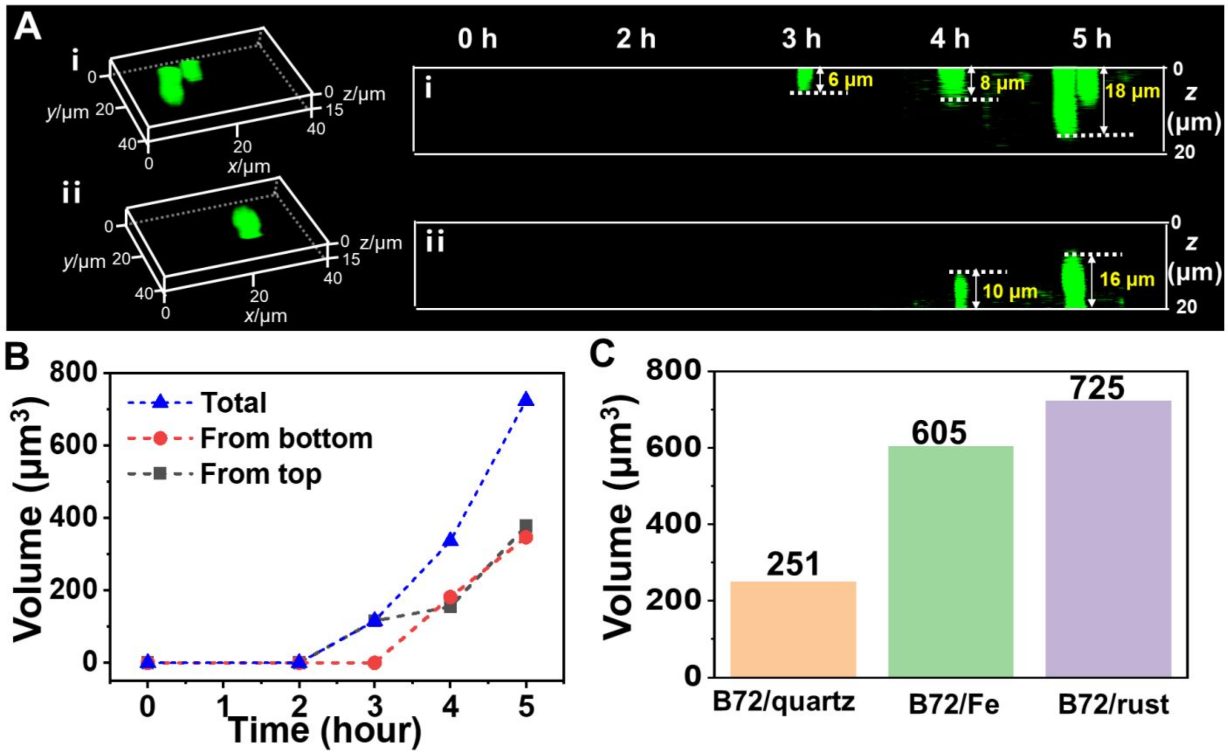

Figure S10. In-situ three-dimensional fluorescence analysis of B72 on the rust. (A) In-situ imaging and (B) quantified volume variations of the B72/rust under photo-thermal treatment. (C) Comparisons of the volume for B72 aged on the different substrates.

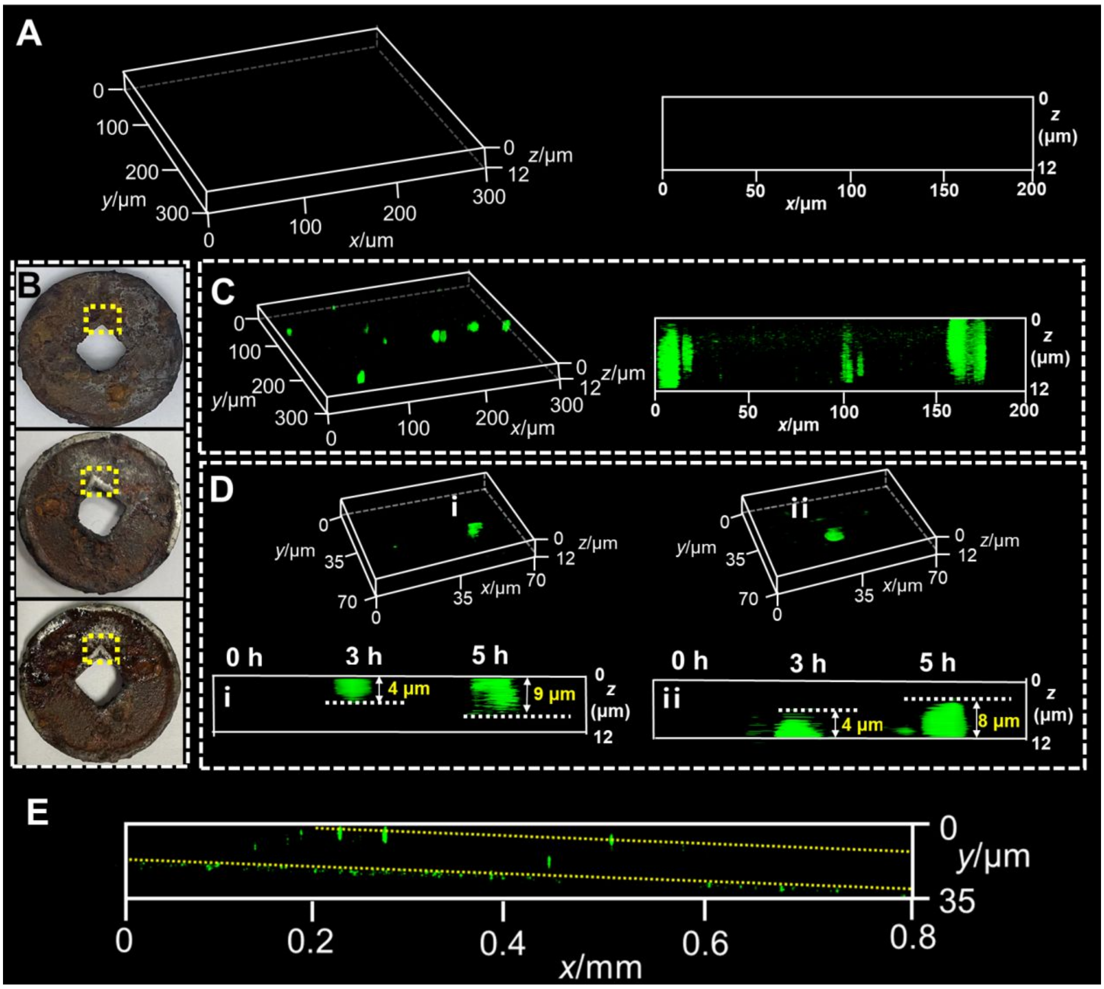

Figure S11. Three-dimensional fluorescence imaging of B72 on real metal artifacts. (A) Three-dimensional and side-view fluorescence image of iron debris from Nanhai No. 1 coated with B72, in the absence of photo-thermal treatment or fluorescence labelling procedures. (B) Photos, (C) three-dimensional and side-view fluorescence images of Northern Song iron coin (from top to bottom. untreated, rust removed, coated with B72 and fluorescent labelled). (D) In-situ monitoring of the aging sites of B72 on Northern Song iron coin. (E) Side-view images of B72 coated on a non-horizontal position of the iron debris from the Nanhai No. 1.

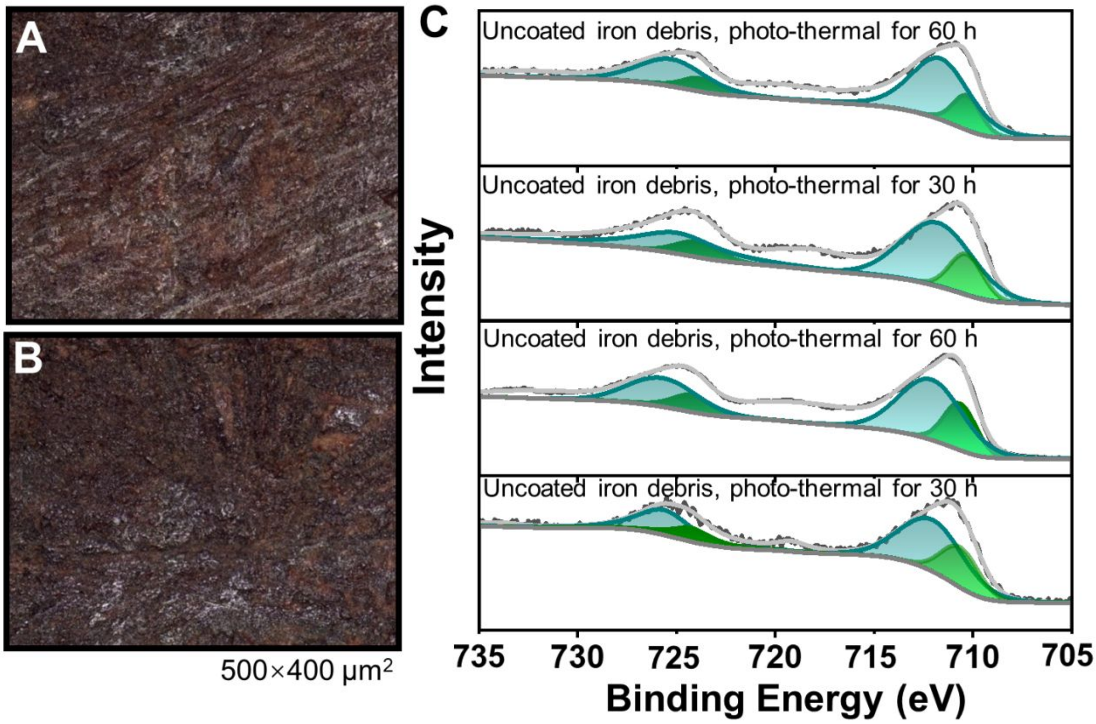

Figure S12. Structural changes of real metal artifact coated with B72. Raman microscopic imaging of Iron debris from Nanhai No. 1 (A) before and (B) after photo-thermal treatment for 90 h (the coated B72 was removed before the Raman measurements). (C) XPS spectra of Fe 2*p* for iron debris from Nanhai No. 1 after photo-thermal treatment for different time (the coated B72 was removed before the XPS measurements).

Table S1. Theoretical and measured concentrations of carboxyl groups in PMAA.

**Theoretical concentration (mol·L^{-1})**	**Measured concentration (mol·L^{-1})**
0.025	0.019
0.050	0.043
0.075	0.065
0.100	0.090
0.125	0.110

Table S2. The fluorescence volume (V) in the x-y-z space calculated from the in-situ fluorescence monitoring for B72/Fe and B72/quartz.

**Samples**	**Fluorescence sites**	**Volume at different aging time (μm³)**				
		0 h	2 h	3 h	4 h	5 h
B72/Fe	"i". from the surface	0	0	71.7	127.5	258.6
	"ii". from the interface	0	102.4	251.4	321.5	346.9
B72/quartz	"i". from the surface	0	55.3	147.5	185.9	251.9
	"ii". from the interface	0	0	0	0	0

Table S3. GPC results for unaged B72 and aged B72 on the different substrates (M_w stood for the weight average molecular weight and M_n represented the number average molecular weight value).

**Samples**	**M_w**	**M_n**
Unaged B72	106802	49897
B72/quartz aged for 30 h	108251	49117
B72/Fe aged for 30 h	105459	62094

Table S4. Fourier transform infrared analysis of unaged B72 and aged B72 on the different substrates.

**Samples**	**1440 cm^{-1}**	**1729 cm^{-1}**	***C.I.***
Unaged B72	0.00914	0.02605	2.85
B72/quartz aged for 30 h	0.02375	0.07150	3.02
B72/Fe aged for 30 h	0.01278	0.05400	4.23

Table S5. Area ratio of C=O/C–C for B72/quartz and B72/Fe before and after photo-thermal aging from XPS measurements.

	**B72/quartz**	**B72/Fe**
0 h	0.08	0.19
30 h	0.13	0.30
Variations	0.05	0.11

Table S6. XPS peak information and result analysis of Fe 2*p* in cast iron.

Samples	Binding energy/eV			Area ratio of [(Fe(III) +Fe(II)) /Fe(0)]
	Fe(III)	Fe(II)	Fe(0)	
Bare Fe, unaged	711.55	709.89	706.99	4.21
Bare Fe, aged for 30 h	711.93	709.90	706.53	7.82
B72/Fe, unaged, remove B72	711.74	710.33	707.01	3.83
B72/Fe, aged for 30 h, remove B72	712.22	710.43	707.16	11.98

Table S7. Current densities (I_{corr}) of the bare cast iron and cast iron coated with B72 before and after aging treatment for 2 days.

I_{corr} (A/cm^2)	Bare Fe	B72/Fe
Unaged	5.16×10^{-4}	1.40×10^{-5}
Aged for 2 days	5.94×10^{-4}	7.31×10^{-5}

Table S8. Corrosion potential (E_{corr}) of the bare cast iron and cast iron coated with B72 before and after aging treatment for 2 days.

E_{corr} (V)	Bare Fe	B72/Fe
Unaged	–0.909	–0.829
Aged for 2 days	–0.913	–0.903

Table S9. Measurements of thickness on the different positions on the iron debris from Nanhai No. 1.

Position	B72/iron debris/mm	Bare iron debris/mm	Thickness of B72/μm
1	2.005	1.993	12
2	1.968	1.954	14
3	1.971	1.959	12
4	1.980	1.969	11
5	2.166	2.155	11
Averaged	–	–	12 ± 2

Table S10. Percentages analysis from Raman scanning mappings for iron rust on Nanhai No. 1 coated with B72 before and after photo-thermal aging treatment.

	**0 h**	**90 h**
Fe_3O_4	1.94	2.78
α-FeOOH	0.20	0.51
β-FeOOH	0.50	0.64
γ-FeOOH	0.00	0.31
C	97.36	95.77

Table S11. Peak information of Fe 2*p* and the varied contents of Fe(III) from XPS spectra for the uncoated iron rust on Nanhai No. 1 after photothermal treatment for different time.

**Aging time (hour)**	**Binding energy/eV**		**Percentage of Fe(III)/%**
	**Fe(III)**	**Fe(II)**	
0	711.73	710.66	71
30	712.04	710.62	70
60	712.11	710.66	73
90	711.92	710.70	71

Table S12. Peak information of Fe 2*p* and the varied contents of Fe(III) from XPS spectra for the B72-coated iron rust on Nanhai No. 1 after photothermal treatment for different time.

Aging time (hour)	Binding energy/eV		Percentage of Fe(III)/%
	Fe(III)	Fe(II)	
0	711.53	710.33	73
30	711.78	710.31	78
60	711.63	710.31	80
90	711.73	710.33	83
